# Mutations in *Dnaaf1* and *Lrrc48* Cause Hydrocephalus, Laterality Defects, and Sinusitis in Mice

**DOI:** 10.1534/g3.116.030791

**Published:** 2016-06-03

**Authors:** Seungshin Ha, Anna M. Lindsay, Andrew E. Timms, David R. Beier

**Affiliations:** * Center for Developmental Biology and Regenerative Medicine, Seattle Children’s Research Institute, Washington 98101; † Division of Genetic Medicine, Department of Pediatrics, University of Washington Medical School, Seattle, Washington

**Keywords:** ENU mutagenesis, motile cilia, *Dnaaf1/Lrrc50/Oda7*, *Lrrc48/FAP134/Drc3*, primary ciliary dyskinesia

## Abstract

We have previously described a forward genetic screen in mice for abnormalities of brain development. Characterization of two hydrocephalus mutants by whole-exome sequencing after whole-genome SNP mapping revealed novel recessive mutations in *Dnaaf1* and *Lrrc48*. Mouse mutants of these two genes have not been previously reported. The *Dnaaf1* mutant carries a mutation at the splice donor site of exon 4, which results in abnormal transcripts. The *Lrrc48* mutation is a missense mutation at a highly conserved leucine residue, which is also associated with a decrease in *Lrrc48* transcription. Both *Dnaaf1* and *Lrrc48* belong to a leucine-rich repeat-containing protein family and are components of the ciliary axoneme. Their *Chlamydomonas* orthologs are known to be required for normal ciliary beat frequency or flagellar waveform, respectively. Some *Dnaaf1* or *Lrrc48* homozygote mutants displayed laterality defects, suggesting a motile cilia defect in the embryonic node. Mucus accumulation and neutrophil infiltration in the maxillary sinuses suggested sinusitis. *Dnaaf1* mutants showed postnatal lethality, and none survived to weaning age. *Lrrc48* mutants survive to adulthood, but had male infertility. ARL13B immunostaining showed the presence of motile cilia in the mutants, and the distal distribution of DNAH9 in the axoneme of upper airway motile cilia appeared normal. The phenotypic abnormalities suggest that mutations in *Dnaaf1* and *Lrrc48* cause defects in motile cilia function.

Motile cilia are important cellular structures for generating fluid flow. Their motility is important for airway mucus clearance, cerebrospinal fluid circulation, leftward extraembryonic fluid flow in the embryonic node, and movement of the sperm and fertilized ovum. Defects in cilia motility can result in human disorders, including primary ciliary dyskinesia (PCD), chronic respiratory tract infection, *situs inversus*, hydrocephalus, and infertility (Badano *et al.* 2006; Fliegauf *et al.* 2007; Brown and Witman 2014; Kurkowiak *et al.* 2015). While many genes have been already associated with human motile cilia defects, genetic diagnostic tests using only currently known causal genes do not explain all cases. For example, causal genes for one third of the PCD cases still remain to be discovered (Kurkowiak *et al.* 2015). Mutations causing motile cilia defects identified in various model organisms are important resources for the discovery of causal genes in human patients, and for understanding their biology.

Hydrocephalus results from an accumulation of the cerebrospinal fluid (CSF) due to obstruction of the ventricular system, abnormal production, circulation, or absorption of CSF. Human patients with hydrocephalus and mouse models share similar characteristics, including enlarged head, ventricular dilation, and damage to the ventricular lining and white matter (McAllister 2012; Lee 2013). Mouse models often display postnatal lethality. A cilial defect is one of the causes for the communicating form of hydrocephalus, and the association of hydrocephalus and PCD has been well known (McAllister 2012; Lee 2013). Ependymal cells lining the ventricles and choroid plexus epithelial cells (CPECs) are the multiciliated cells in the brain (Lee 2013; Narita and Takeda 2015). In mice, motile cilia on ependymal cells form postnatally and generate directional CSF flow. CPECs undergo ciliogenesis during midgestation in mice and produce CSF. Cilia on CPECs are immotile but transiently motile during the perinatal period. An ependymal cell cilia defect resulting in abnormal CSF circulation is considered the primary mechanism for cilia-related hydrocephalus, but the association of a CPEC cilia defect with neonatal hydrocephaly in mice has been also suggested (Banizs *et al.* 2005; Lee 2013; Narita and Takeda 2015).

Abnormal ultrastructure of the ciliary axoneme is a hallmark of a motile cilia defect. The axoneme of motile cilia has a 9 + 2 structure, with an exception of nodal cilia that have 9 + 0 organization (Fliegauf *et al.* 2007). The 9 + 2 axoneme has nine peripheral microtubule doublets (A and B tubules) and two central single microtubules (central pair), while the 9 + 0 axoneme lacks the central pair. The inner and outer dynein arms (IDA and ODA) extend from the A-tubules toward the B-tubules of neighboring doublets, and are responsible for ciliary movement generation. The A-tubules and adjacent B-tubule of neighboring doublets are linked by the nexin-dynein regulatory complex (N-DRC), which is a conserved structure from algae to humans. It has been proposed that the N-DRC is important for the coordination of microtubule sliding by maintaining microtubule doublet alignment during flagella bending (Porter and Sale 2000; Nicastro *et al.* 2006; Heuser *et al.* 2009; Bower *et al.* 2013). The peripheral microtubules are connected to the central pair by the radial spokes. Mutations in many axonemal structural component genes, including ODA, IDA, and N-DRC, are linked to human motile cilia disorders (Knowles *et al.* 2013; Kurkowiak *et al.* 2015).

We have reported previously the identification and mapping of two hydrocephalus mutant lines, described as line 42 and 67, which we ascertained in a forward genetic screen for abnormal brain phenotypes (Ha *et al.* 2015). In this paper, we report that these lines carry mutations in *Dnaaf1* (*Dynein* *axonemal* *assembly factor 1*) and *Lrrc48* (*Leucine-rich repeat-containing protein 48*), respectively. Both of these genes are previously known to encode structural components of the motile cilial axoneme. *Dnaaf1*, also known as *Lrrc50* (*Leucine-rich repeat-containing protein 50*) or *Oda7* (*Outer dynein arm 7*), is important for ciliary beat frequency in *Chlamydomonas reinhardtii* and a causal gene for primary ciliary dyskinesia (PCD) in humans (Kamiya 1988; Freshour *et al.* 2007; Duquesnoy *et al.* 2009; Loges *et al.* 2009). The *Chlamydomonas* ortholog of *Lrrc48* is *FAP134 (Flagellar associated protein 134) or Drc3 (Dynein regulating complex 3)*, which is a component of the N-DRC and required for normal flagellar waveform (Pazour *et al.* 2005; Lechtreck *et al.* 2009; Lin *et al.* 2011; Bower *et al.* 2013; Awata *et al.* 2015). Of note, mouse mutants of *Dnaaf1* and *Lrrc48* have not been previously reported. In addition to hydrocephalus, both lines demonstrate laterality defects and sinusitis, which is consistent with the presence of a presumptive motile cilia defect.

## Materials and Methods

### Animals and genotyping

Generation of *Dnaaf1^m4Bei^* and *Lrrc48^m6Bei^* mutants was previously described (Ha *et al.* 2015). *Dnaaf1^m4Bei^* was previously named line 42, and is currently listed as m4Bei (MGI:5505439) in the Mouse Genome Informatics (MGI) database. *Lrrc48^m6Bei^* was previously named line 67 and is listed as m6Bei (MGI:5505443) in MGI. The mutant lines originated from mutagenized A/J (Jackson Laboratory) and were maintained by serial backcrosses to C57BL6/N (Taconic) females. Genotyping was performed using microsatellite markers and Metaphor agarose (Lonza) gel electrophoresis to identify an A/J region derived from the original mutagenized parent, or using Sanger sequencing to detect the mutations directly. The primer sequences for genotyping are listed in Supplemental Material, Table S1. Animals were maintained in accordance with the guidelines of the National Institutes of Health and the Seattle Children’s Research Institute’s Institutional Animal Care and Use Committee.

### Exome sequencing

Genomic DNAs were prepared using a DNeasy kit (Qiagen) from the liver tissue of hydrocephalus mutants. Whole-exome sequencing was performed to analyze the entire coding and splice-junction sequence at the Broad Institute (Cambridge, MA) as a part of the Mouse Mutant Resequencing project (https://www.broadinstitute.org/mouse-mutant-resequencing). Reads were mapped to the mouse genome mm10 using BWA-MEM (Li and Durbin 2009), and duplicate reads marked using picard (http://broadinstitute.github.io/picard). Subsequent processing and variant calling was completed using the Genome Analysis Toolkit (GATK) (McKenna *et al.* 2010). The average coverages were 65X and 72X of the exome for *Dnaaf1^m4Bei^* and *Lrrc48^m6Bei^*, respectively, with > 91.4% of the exome covered by 10 or more reads. To identify the causal mutations, we considered all nonsynonymous, nonsense, or splicing mutations within recombinant intervals defined by SNP analysis. The *Dnaaf1^m4Bei^* and *Lrrc48^m6Bei^* lines each had only one homozygous variant that fitted these criteria.

### RT-PCR and qRT-PCR

Total RNA was isolated from the brains of wild-type and mutant mice using Trizol reagent (Sigma). A Superscript III First-Strand RT-PCR kit (ThermoFisher Scientific) was used for cDNA synthesis following the manufacturer’s instructions, using the same amount of RNA. RT-PCR was performed using the cDNA and primers indicated in Table S2, designed using Primer3plus (http://www.bioinformatics.nl/cgi-bin/primer3plus/primer3plus.cgi). Primer pairs for qRT-PCR were designed using Primer-BLAST (http://www.ncbi.nlm.nih.gov/tools/primer-blast) and tested to determine their specificity, optimal reaction temperature, melt curve/peak, and efficiency. qRT-PCR was performed using KAPA SYBR FAST qPCR Kits (KAPA Biosystems) and the CFX96 Real-Time System with a C1000 Thermal Cycler (BioRad). cDNAs from four mice for each genotype (wild-type and homozygote mutant) were used, and the reactions for each sample were triplicated. Mean Cq values for each sample were calculated from the triplicate measurement, *Lrrc48* expression was normalized to *Actb*, relative expression levels compared with the wild-type were calculated, and statistical analysis was performed using REST (Relative Expression Software Tool, http://rest.gene-quantification.info). Power analysis was performed using PS (Power and Sample size calculation software, http://biostat.mc.vanderbilt.edu/wiki/Main/PowerSampleSize) to determine sample size, and four samples were enough to reject the null hypothesis with probability 0.9.

### Analysis of Dnaaf1 splicing defect

RT-PCR products from the wild-type and *Dnaaf1^m4Bei^* mutant were cloned into the pCR2.1 vector using a TOPO TA cloning kit (Invitrogen). Plasmid DNAs were isolated from single colonies, and wild-type and mutant clones were screened by PCR and sequenced using M13 forward and reverse primers. Selected clones with insert sizes similar to the major RT-PCR bands were amplified using the same primers (*Dnaaf1* e3-6, Table S2), and the sizes of PCR products were compared to the RT-PCR products by electrophoresis.

### Histology

For Nissl staining, the brains were fixed in Bouin’s fixative for 24 hr and then in 10% phosphate-buffered formalin overnight. The fixed brains were embedded in paraffin and sectioned using a Leica microtome. Sections 10-μm thick were stained using FD Thionin solution (FD NeuroTechnologies) following the manufacturer’s instruction.

For hematoxylin and eosin staining of the maxillary sinuses, the whole heads were fixed in Bouin’s fixative and decalcified using Poly-NoCal solution (Polysciences). After 4 hr (P7 mice) or 8 hr (16 week-old mice) incubation, endpoint of decalcification was determined using a Poly-NoCal endpoint determination kit (Polysciences). The decalcified heads were processed, embedded in paraffin, sectioned 10 μm in thickness, and then hematoxylin and eosin staining was performed. Imaging of histology slides was done using a Leica DM4000B upright microscope and DFC310FX camera.

### Immunohistochemistry

The tissues were fixed in 4% PFA in PBS overnight, incubated in 30% sucrose until they sank, and then embedded in OCT compound. Sections 10-μm thick were mounted on the glass slides and immunostaining was performed. The brain sections were incubated with blocking solution (3% goat serum, 3% bovine serum albumin, 0.3% Triton X-100 in PBS) for 2 hr, with mouse anti-ARL13B (NeuroMab, clone N295B/66, 1:200) and rabbit anti-dynein heavy chain (DNAH9) antibody (Abcam, ab133968, 1:200) overnight at 4°, with the secondary antibodies (Alexa Fluor 488 goat anti-mouse IgG2a and Alexa Fluor 568 goat anti-rabbit IgG) for 2 hr at room temperature, and then with Hoechst for 5 min. The immunostained sections were imaged using Leica TCS SP5 or Zeiss LSM710 confocal microscopes.

### Data availability

DNA sequence data is available in the SRA sequence archive using the accession number: SRP076681. Otherwise, all data necessary for confirming the conclusions presented in the article are represented fully within the article.

## Results

### Identification and analysis of Dnaaf1 and Lrrc48 mutations

The originally reported recombination intervals based on mouse genome assembly version mm9 were chr8:105–130 Mb for line 42 and chr11:47–69 Mb for line 67 (Ha *et al.* 2015). Mb positions used in this paper are from mm10 unless indicated otherwise, and SNPs flanking the original recombinant interval and updated map positions are as follows: rs3705275 (chr8:103,336,314 bp) and rs6400423 (chr8:126,582,425 bp) in line 42, and rs13481014 (chr11:48,117,382 bp) and rs13481084 (chr11:68,770,517 bp) in line 67. Whole-exome sequencing was performed to identify recessive mutations within the homozygous regions previously defined using whole-genome SNP panels (Ha *et al.* 2015). Mutations in *Dnaaf1* and *Lrrc48* were the only nonsynonymous or splice-site homozygous variants found in the recombinant intervals defined by SNP analysis.

We determined that the mutant mice used for exome sequencing included recombinants within the original interval; therefore, additional analysis of the exome sequencing data was done to identify a minimal region carrying homozygous A/J strain-specific SNPs. We have previously described a detailed method of mutant mapping utilizing exome sequencing data (Gallego-Llamas *et al.* 2015). This further narrowed the interval to chr8:119.4–129.4 Mb for line 42 and chr11:54.1–63.8 Mb for line 67. This increased resolution excluded some candidate genes; for example, *Dnah2* at chr11:69,420,809–69,549,108 bp. Lists of genes in the newly defined interval and exome sequencing coverage are shown in Figure S1. Importantly, the histologically-confirmed hydrocephalus phenotype has remained 100% concordant with homozygosity for the mutations in *Dnaaf1* (*N* = 12) and *Lrrc48* (*N* = 16), and we have never observed hydrocephalus in mice that are not homozygous at these loci (*N* > 100 for both lines).

**Figure 1:**
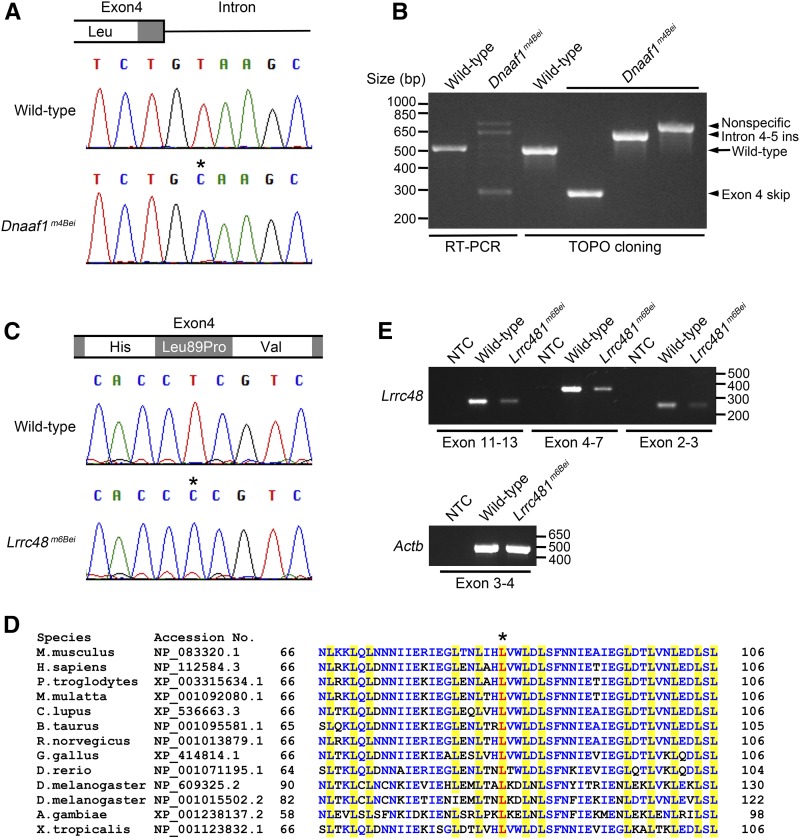
Mutations in *Dnaaf1* and *Lrrc48*. (A) A chromatogram from Sanger sequencing showing a T > C mutation (asterisk) at the junction of exon 4 and intron. (B) RT-PCR result showing amplification of a fragment corresponding to exon 3–6 of *Dnaaf1* (lanes 1 and 2). Multiple bands with incorrect sizes were detected in *Dnaaf1^m4Bei^*, indicating a splicing defect. Cloned RT-PCR products revealed abnormal transcripts with exon 4 skipping (lane 4, insert size 279 bp) or intron 4–5 insertion (lane 5, insert size 617 bp). The largest band (nonspecific) was not a *Dnaaf1* transcript. (C) A chromatogram from Sanger sequencing showing a T > C mutation (asterisk) causing an amino acid change (Leu89Pro). (D) Multiple protein sequence alignment adapted from NCBI HomoloGene search result, including conserved leucine-rich repeat domains near the mutation. Leucine residues in the domain are highlighted in yellow. The leucine residue substituted in *Lrrc48^m6Bei^* mutants is shown in red (asterisk). Other conserved residues are marked in blue. (E) RT-PCR results using three different primer sets spanning the *Lrrc48* transcript. All amplified weaker bands from the mutant. *Actb* (*Beta-actin*) was used as a control. NCBI, National Center for Biotechnology Information; NTC, no template control; RT-PCR, reverse transcription polymerase chain reaction.

Line 42 had a mutation in *Dnaaf1* (NM_026648) at chr8:119582732 bp (exon 4: c.556 + 2T > C). This is in the splice donor site of exon 4, two bases distal from the exon (Figure S2A). The mutant line is referred to *Dnaaf1^m4Bei^* in this paper, as listed in the MGI database. Sanger sequencing analysis was performed to confirm the mutation (Figure 1A). The mutation at the splice donor site suggested the likelihood of a splicing defect, therefore the *Dnaaf1* transcript was analyzed by RT-PCR using primers designed to amplify a fragment spanning exon 3 and exon 6. The wild-type mice showed a band at expected size 501 bp, while multiple bands with incorrect sizes were detected in the *Dnaaf1^m4Bei^* mutant (Figure 1B). This result confirmed that the *Dnaaf1^m4Bei^* mutation causes abnormal splicing. To examine abnormal transcripts, RT-PCR products were cloned into a plasmid vector, and individual clones were analyzed using PCR and sequencing (Figure 1B). No abnormal *Dnaaf1* transcripts were obtained from the wild type. In contrast, 48 clones from a *Dnaaf1^m4Bei^* mutant were analyzed and no normal transcript of 501 bp was found. Eleven mutant clones had 222 bp of the entire exon 4 skipped (Figure 1B, c.335_556del). Five mutant clones retained the entire intron 4–5 between exon 4 and 5 (Figure 1B, [c.556 + 2T > C + c.556_557ins556 + 1_556 + 116]). The remainder of clones had sequences unrelated to *Dnaaf1*.

Transcripts with exon 4 skipped would encode a DNAAF1 protein with an in-frame deletion of 74 amino acids (p.G112_S186delinsA). *Dnaaf1* is a leucine-rich repeat-containing protein (Duquesnoy *et al.* 2009), and its predicted protein domain structures are shown in Figure S3A. Exon 4 deletion will remove three out of six leucine-rich repeats. Intron 4–5 insertion introduces a S186C substitution at the junction with the addition of 43 amino acids followed by a premature stop codon (p.S186CfsX43), which would result in a large truncation of 448 C-terminal amino acids.

A mutation in *Lrrc48* (NM_029044) at Chr11:60363569 bp (exon4: c.T266C: p.L89P) was found in line 67 by the whole-exome sequencing analysis (Figure S2B). This is a missense mutation that substitutes proline for leucine. The mutant line is referred to as *Lrrc48^m6Bei^* in this paper, as listed in the MGI database. A chromatogram from Sanger sequencing analysis is shown in Figure 1C. *Lrrc48* is also a leucine-rich repeat-containing protein (Figure S3B), and the mutation alters a highly-conserved leucine residue (Figure 1D) located within the leucine-rich repeat domain (Bella *et al.* 2008). Importantly, the PolyPhen score for this missense mutation is 1.0 (Adzhubei *et al.* 2010). In addition, analysis of the *Lrrc48* transcript using three independent primer sets suggested that the mutant mice have decreased expression (Figure 1E). qRT-RCR analysis using primers for exon 8–9 confirmed a reduction of *Lrrc48* expression in the mutant to 34% of wild-type levels (*P* < 0.001, Figure S4).

### Hydrocephalus

**Figure 2:**
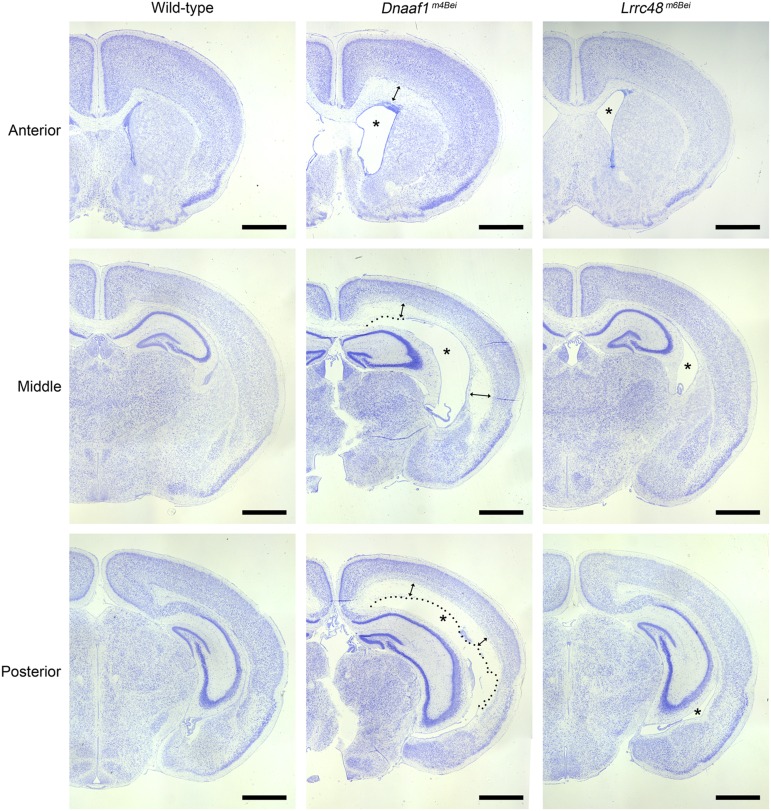
Hydrocephalus phenotype. Nissl-stained images of coronal sections are shown. Enlarged lateral ventricles are marked with asterisks. Enlargement of the third ventricles is also evident in both mutants. Dotted lines in *Dnaaf1^m4Bei^* mutant images indicate disruption in ependymal lining. Double-headed arrows indicate transependymal edema. Scale bars, 1 mm.

Both mutant lines were originally ascertained as having hydrocephalus prior to P21 (Ha *et al.* 2015). At birth, both *Dnaaf1^m4Bei^* and *Lrrc48^m6Bei^* mutant brains appeared grossly normal. The lateral ventricle size, aqueduct opening, and choroid plexus morphology were comparable to wild-type littermates. The lack of aqueduct stenosis at birth suggests that the mutants have a communicating form of hydrocephalus or ventriculomegaly, although we did not exclude the possibility that obstructive hydrocephalus develops secondarily afterward. At postnatal day 10 (P10), changes in the brain structure are already evident (Figure 2). The lateral ventricles and third ventricle were significantly enlarged. In mutants with severe hydrocephalus, the ependymal lining was disrupted, and transependymal edema of the white matter was noticeable. *Dnaaf1^m4Bei^* mutants usually displayed more severe hydrocephalus compared to *Lrrc48^m6Bei^*.

### Postnatal lethality of Dnaaf1^m4Bei^

Most of the *Dnaaf1^m4Bei^* mutants were severely runted compared with littermates (Figure S5) and frequently died during the second postnatal week. Postnatal lethality is described in Table 1. Many homozygotes (17.1% of total pups and 68.3% of expected homozygote pups) survived at P1, while only 8.6% of total pups and 34.3% of expected homozygote pups survived at P10. Furthermore, no homozygote was recovered at weaning (P21) when 33 mice from six litters born from heterozygote mating pairs were genotyped (Chi-square test, *P* = 0.0037). Although similarly severe hydrocephalus or runting was occasionally observed in litters born from *Lrrc48^m6Bei^* heterozygote mating pairs, most *Lrrc48^m6Bei^* homozygotes were able to survive to adulthood. A total of 71 mice from 11 litters were genotyped, and 18 (25.4%) were homozygotes.

**Table 1:** Postnatal lethality and laterality defect

Age	P0–P1*^a^*		P10		P21	
*Dnaaf1^m4Bei^*	Observed*^b^*	Expected	Observed*^c^*	Expected	Observed*^d^*	Expected
Wild-type	29	20.5	21	17.5	12	8.25
Heterozygote	39	41	43	35	21	16.5
Homozygote	14	20.5	6	17.5	0	8.25
Laterality defect	8 (57.1%)		2 (33.3%)		N/A*^e^*	
* Situs inversus*	3		0			
Heterotaxy	5		2			
*Lrrc48^m6Bei^*	Observed	Expected	Observed	Expected	Observed	Expected
Wild-type	21	18	24	19.75	22	17.75
Heterozygote	33	36	36	39.5	31	35.5
Homozygote	18	18	19	19.75	18	17.75
Laterality defect	2 (11.1%)		1 (5.3%)		N/A*^e^*	
* Situs inversus*	2		0			
Heterotaxy	0		1			

N/A, not applicable.

a Only one litter was P0 out of total 11 litters.

b Chi-square test, *P* = 0.0583 (two-tailed).

c Chi-square test, *P* = 0.0065 (two-tailed).

d Chi-square test, *P* = 0.0037 (two-tailed).

e Laterality defect was not examined at P21.

### Male infertility of Lrrc48^m6Bei^

We observed during mouse husbandry that male *Lrrc48^m6Bei^* homozygotes never generated pups, while homozygote females were fertile. To confirm the male infertility phenotype, six homozygote males between the age of 8–14 wk were set up with four different wild-type females. All six males were able to plug at least one female, and a total of nine out of 24 females were plugged. However, none of the females became pregnant.

### Laterality defect

**Figure 3:**
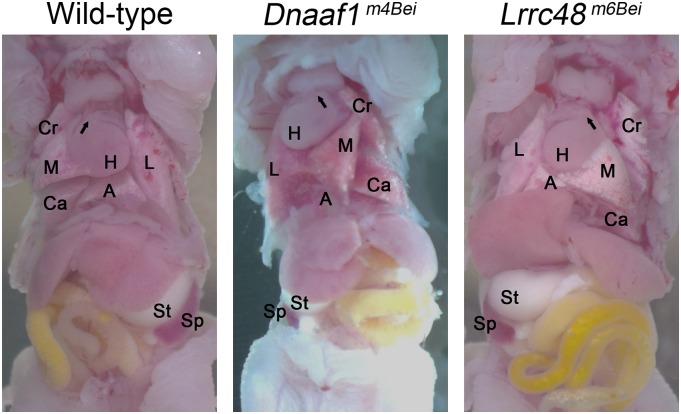
*Situs inversus* phenotype. The position of the heart (H), lung lobes (L, Cr, M, Ca, and A), stomach (St), and spleen (Sp) shows a laterality defect. Arrows indicate the angle of pulmonary trunk. A, accessary lobe; Ca, right caudal lobe; Cr, right cranial lobe; L, left lobe; M, right middle lobe.

To assess if the mutants have a laterality defect, the visceral organs were examined at P1 and P10. Mutants with *situs inversus* were found from both *Dnaaf1^m4Bei^* and *Lrrc48^m6Bei^* mutant lines (Figure 3). The numbers of mutants with laterality defects, including *situs inversus* and heterotaxy, are shown in Table 1. *Dnaaf1^m4Bei^* mutants express laterality defects more frequently than *Lrrc48^m6Bei^* mutants. Combining observations at P1 and P10, 52.6% and 8.1% of homozygotes from *Dnaaf1^m4Bei^* and *Lrrc48^m6Bei^* mutant lines, respectively, showed laterality defects.

### Sinusitis

**Figure 4:**
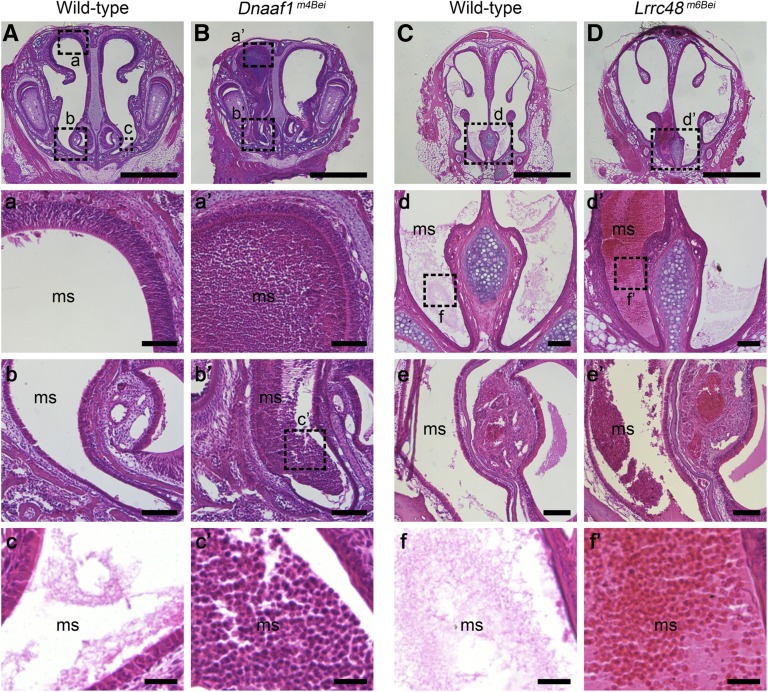
Mucus accumulation in the sinuses. (A–D) Coronal sections of the maxillary sinuses (ms) from mutant and littermate wild-type mice are stained using hematoxylin and eosin. Higher magnification images of the boxed region (a–f for wild-type and a’–f’ for mutants) are shown in lower panels. e and e’ are from more posterior paranasal cavity sections. Severe mucus accumulation and infiltration of neutrophils (c’) were obvious in a *Dnaaf1^m4Bei^* mutant at P7. *Lrrc48^m6Bei^* mutants show milder phenotype that is undetectable until later (16 week-old). Scale bars: A–D, 1 mm; a, a’, b, b’, d’, e, and e’, 100 μm; c, c’, f, and f’, 25 μm.

As sinusitis is a common feature of human PCD, histological analysis of maxillary sinuses was performed (Figure 4). Both *Dnaaf1^m4Bei^* and *Lrrc48^m6Bei^* mutants showed similar phenotypes to those reported for other mouse PCD models (Ibanez-Tallon *et al.* 2002; Kobayashi *et al.* 2002; Sironen *et al.* 2011; Lucas *et al.* 2012); this included mucus accumulation and infiltration of neutrophils. *Dnaaf1^m4Bei^* mutants displayed a severe phenotype at P7, while *Lrrc48^m6Bei^* mutants displayed a mild phenotype at older age (16 wk).

### Examination of the motile cilia

**Figure 5:**
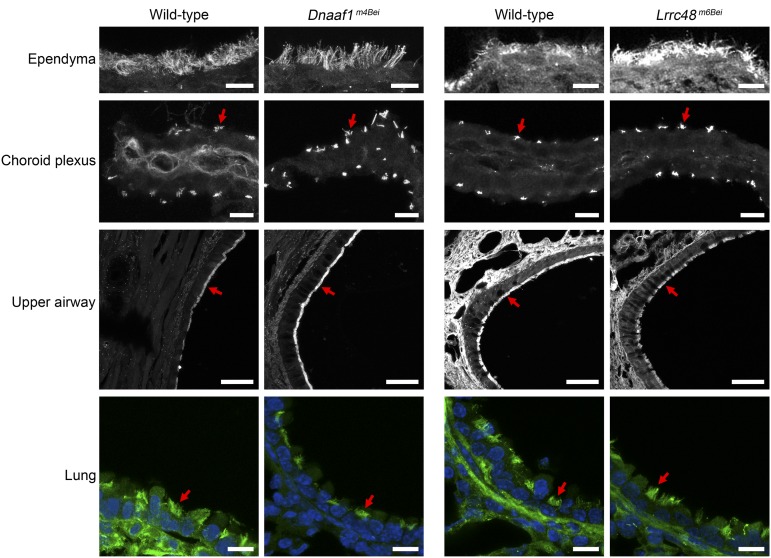
The presence of cilia in the mutants. Cilia in ependyma and choroid plexus of the brain, upper airway, and lung were visualized by anti-ARL13B immunostaining (gray or green). The nuclei were stained using Hoechst (blue). Red arrows indicate representative cilia. Scale bars: ependyma, choroid plexus, and lung, 10 μm; upper airway, 50 μm.

**Figure 6:**
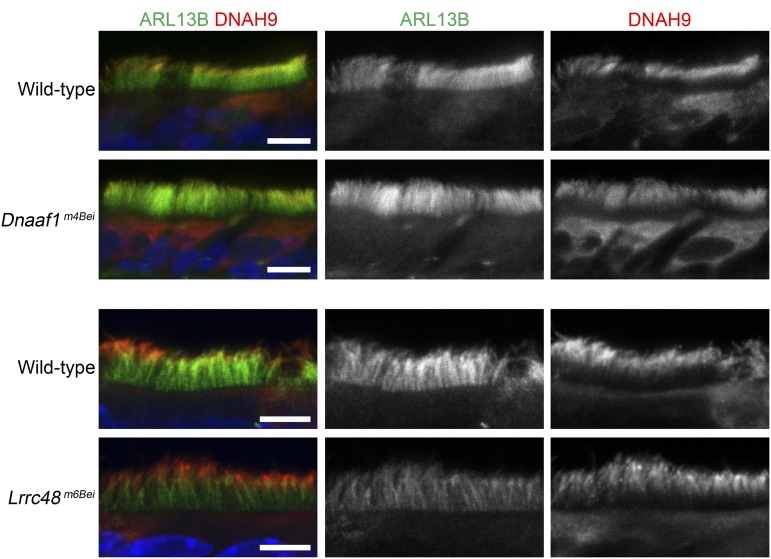
Normal localization of DNAH9 in the mutants. Anti-ARL13B (green, middle) and anti-DNAH9 (red, right) coimmunostaining was performed. DNAH9 is distally localized in the ciliary axoneme of the motile cilia in the upper airway. The nuclei were stained using Hoechst (blue). Scale bars, 5 μm.

We examined the motile cilia using ARL13B as a marker and did not find any obvious abnormalities; the cilia were present in the ependyma and choroid plexus of the lateral ventricles, upper airway, and bronchi of the lung (Figure 5). It has been previously shown that outer dynein arm heavy chain components, including DNAH9, are absent in the motile cilia of primary respiratory cells from human patients carrying *DNAAF1/LRRC50* mutations (Loges *et al.* 2009). We performed coimmunostaining of ARL13B and DNAH9 on the upper airway sections and found that DNAH9 was present and normally localized in the motile cilia of mutant mice (Figure 6).

## Discussion

Murine forward genetic screens for developmental phenotypes have generated numerous mutants with cilial defects. Interestingly, genes encoding cilial proteins have been identified relatively frequently from multiple independent developmental screens, regardless of whether the screen was specifically targeting laterality defects, embryonic lethality, organogenesis defects, or discrete phenotypes such as congenital heart defects or brain patterning phenotypes, indicating a crucial role of cilia during the development of multiple organ systems (Kasarskis *et al.* 1998; Tran *et al.* 2008; Ermakov *et al.* 2009; Rao Damerla *et al.* 2014; Ha *et al.* 2015; Li *et al.* 2015). Proteomic analyses of cilia have revealed the presence of a surprisingly large number of proteins despite the small size of this organelle (Ostrowski *et al.* 2002; Pazour *et al.* 2005; Ishikawa *et al.* 2012), and new genes required for cilial function are still being discovered. Of particular note is a recent comprehensive screen for congenital heart disease, which revealed an enrichment of mutations associated with cilial function and laterality defects (Ermakov *et al.* 2009; Li *et al.* 2015),

In this paper, we report the characterization of two novel mouse models of cilial defects, *Dnaaf1^m4Bei^* and *Lrrc48^m6Bei^*, which both display hydrocephalus and laterality defects. The *Dnaaf1^m4Bei^* mutant carries a splice site mutation that affects transcription with high penetrance; the abnormal transcripts that are generated would result in either a large internal deletion or premature protein truncation. The *Lrrc48^m6Bei^* mutant carries a missense mutation in a highly conserved leucine-rich repeat. This mutant also shows a reduction in expression; however, as mice that are heterozygous for null mutations of genes required for motile ciliary function appear normal (*e.g.*, Tan *et al.* 2007), it seems unlikely that the reduced gene expression accounts for the mutant phenotype.

Both genes are known to encode proteins required for motile cilia and flagellar function; however, mouse mutants of *Dnaaf1* and *Lrrc48* have not been previously reported. Despite the presence of motile cilia and the normal localization of DNAH9 in these mutants, their phenotypes suggest defects in motile cilia function. Considering what is already known about the *Chlamydomonas* orthologs of these genes, as discussed below, it is possible that mutations in mice may cause abnormalities in axonemal ultrastructure and cilia motility, rather than a ciliogenesis defect. Further analysis of the axonemal ultrastructure and ciliary motility might illuminate the nature of motile cilia dysfunction in *Dnaaf1^m4Bei^* and *Lrrc48^m6Bei^* mutants.

*Lrrc48* has been previously described as an ortholog of the *C. reinhardtii* gene *Flagella Associated Protein (FAP) 134* (Pazour *et al.* 2005; Lechtreck *et al.* 2009; Lin *et al.* 2011; Bower *et al.* 2013). FAP134 protein was isolated from the flagellar proteome and its localization in the flagellar axoneme has been confirmed (Pazour *et al.* 2005; Lechtreck *et al.* 2009). Another peptide sequencing analysis identified FAP134 to be DRC3 (Lin *et al.* 2011). DRC3 protein is one of the eleven known components of N-DRC. DRC3 was originally identified by a proteomics analysis of flagellar axonemal polypeptides, using a series of flagella motility mutants (Huang *et al.* 1982; Piperno *et al.* 1992; Lin *et al.* 2011; Bower *et al.* 2013).

More specifically, FAP134/DRC3 was found to be absent or reduced in the flagellar axoneme of *Chlamydomonas* mutants *pf2* and *sup-pf-3* (Bower *et al.* 2013). These mutant strains are suppressor strains that reverse flagellar immotility in paralysis mutant strains lacking the central microtubule pairs (Huang *et al.* 1982). However, *pf2* and *sup-pf-3* do not carry mutations in the FAP134/DRC3 gene, but in the DRC4 and ODA2 genes, respectively (Rupp *et al.* 1996; Rupp and Porter 2003). They display an overall structural defect of N-DRC involving multiple N-DRC components; as such, the flagellar motility defect observed in *pf2* and *sup-pf-3* is not direct evidence to prove the requirement of FAP134/DRC3. More recently, the *drc3* mutant was reported to display slower swimming speed due to an abnormal flagellar waveform (Awata *et al.* 2015). This mutant, generated by an insertional mutagenesis, has a large deletion including the first seven exons of FAP134/DRC3. All previously known N-DRC components were found present in the axoneme of the *drc3* mutant, providing direct evidence for the importance of FAP134/DRC3 in flagellar motility (Awata *et al.* 2015).

Our *Lrrc48^m6Bei^* mutant phenotype, as the first reported mouse mutant of the *FAP134/DRC3* ortholog, demonstrates a presumptive requirement for this gene in mammalian motile cilial function. Consistent with the observation that *Chlamydomonas* N-DRC component mutants have no change in flagellar length (Piperno *et al.* 1992; Awata *et al.* 2015), ciliogenesis was normal in our mouse mutants. We did not pursue further analysis of axonemal ultrastructure as it requires high-resolution *in situ* cryo-electron tomography. A study using this technique has recently visualized DRC3 localization to the L1 protrusion of the nexin linker region of N-DRC and its direct interaction with the dynein g motor domain of an inner dynein arm, IA5 (Heuser *et al.* 2009; Awata *et al.* 2015; Song *et al.* 2015). The *drc3* mutant displayed a mild alteration of N-DRC structure restricted to a small portion near DRC3, unlike other N-DRC component mutants (Awata *et al.* 2015). This type of ultrastructural defect would not be easily detectable using conventional transmission electron microscopy.

*Dnaaf1* is also called *Lrrc50* (human and zebrafish) or *Oda7* (*Chlamydomonas reinhardtii*), and its function is relatively well characterized. In *Chlamydomonas*, a genomic deletion that included exon 1 of the *Oda7* gene was identified in an *arg7*, *oda7* mutant strain (Freshour *et al.* 2007). This strain was ascertained in a screen for slow-swimming phenotypes with reduced flagellar beat frequency, and the entire outer dynein arm (ODA) was absent in many of these mutants (Kamiya and Okamoto 1985). Analysis of axonemal ultrastructure revealed that the *Oda7* mutant lacks any observable pool of outer row dynein heavy chain α (Fowkes and Mitchell 1998). Freshour *et al.* (2007) found that ODA7 interacts with both outer row and I1 inner row dyneins, and that the mutation in ODA7 prevents axonemal outer row dynein assembly by blocking the cytoplasmic assembly of heavy chains and intermediate chains.

In zebrafish, the *lrrc50^hu255H^* mutant carrying a nonsense mutation at a conserved leucine residue was identified from ENU mutagenesis (van Rooijen *et al.* 2008). This mutant displayed an ultrastructural defect of the ciliary axoneme, including the absence of ODA and misalignments of outer microtubule doublets. A range of motile cilia defects in multiple organs were observed: the absence of motile cilia in the nose and neural tube, irregular cilia distribution in Kupffer’s vesicle, abnormal cilia morphology and distribution in the anterior and posterior pronephric duct, reduced kinocilium number and length in the lateral line organ, and reduced cilia motility measured by dye-excretion test. As a result, the mutant has a laterality defect and develops pronephric tubular cysts.

Importantly, both *Dnaaf1* and *Lrrc48* mutations have a clinical relevance. *DNAAF1* mutations have been found in PCD patients with or without *situs inversus* (Duquesnoy *et al.* 2009; Loges *et al.* 2009) and seminoma patients (Basten *et al.* 2013). Analysis of the axonemal ultrastructure in human PCD patients revealed the absence of both dynein arms (Duquesnoy *et al.* 2009; Loges *et al.* 2009). It is likely that *Dnaaf1^m4Bei^* mutants have a similar axonemal ultrastructure defect, given that ODA structure is conserved from algae to zebrafish to human. *DNAAF1* is included in genetic diagnostic testing of PCD (Knowles *et al.* 2013; Kurkowiak *et al.* 2015; Lobo *et al.* 2015; Marshall *et al.* 2015). In contrast, the direct clinical relevance of *LRRC48* mutation has not yet been reported. However, orthologs of multiple N-DRC component genes have been implicated in human PCD, including *CCDC164/DRC1* and *CCDC65/DRC2* (Austin-Tse *et al.* 2013; Horani *et al.* 2013; Wirschell *et al.* 2013). It was found that LRRC48 protein, which is normally localized in the axoneme, was reduced in PCD patients with *CCDC164/DRC1* mutation (Wirschell *et al.* 2013).

In summary, we report the phenotypic and genomic characterization of mice carrying mutations in *Dnaaf1* and *Lrrc48*, which are presumptively required for the function of primary motile cilia. For unknown reasons, mouse models of PCD frequently display hydrocephalus, which is less common in humans than other PCD symptoms such as respiratory infection and *situs inversus*. In addition to differences in shape and size, human and mouse brains have structural and physiological differences that may influence their susceptibility to PCD-associated hydrocephalus (reviewed in Lee 2013). Despite this, it is likely that the underlying physiology of motile cilial function is conserved across species; as such, the *Dnaaf1^m4Bei^* and *Lrrc48^m6Bei^* mutants can serve as valuable models of human PCD.

## Supplementary Material

Supplemental Material
