# Supplementary material for: Mutations in Dnaaf1 and Lrrc48 Cause Hydrocephalus, Laterality Defects, and Sinusitis in Mice
Source: G3 (Bethesda). 2016 Jun 3;6(8):2479–87. doi: 10.1534/g3.116.030791 (PMC4978901; doi:10.1534/g3.116.030791)
Supplement: Supplemental Material [file supp_6_8_2479__index.html]

Mutations in Dnaaf1 and Lrrc48 Cause Hydrocephalus, Laterality Defects, and Sinusitis in Mice — Supplemental Material 

# Mutations in *Dnaaf1* and *Lrrc48* Cause Hydrocephalus, Laterality Defects, and Sinusitis in Mice

## Supplemental Material for Ha *et al.*, 2016

**Files in this Data Supplement:**

- Figure S1 - Lists of genes aligned against exome sequencing coverage. (.pdf, 1 MB)
- Figure S2 - Exome sequencing results. (.pdf, 668 KB)
- Figure S3 - Protein domain structures from NCBI conserved domain search. (.pdf, 170 KB)
- Figure S4 - qRT-PCR analysis of *Lrrc48*. (.pdf, 461 KB)
- Figure S5 - *Dnaaf1 m4Bei* homozygotes are runted. (.pdf, 589 KB)
- Table S1 - Primers used for genotyping. (.pdf, 69 KB)
- Table S2 - Primers used for RT-PCR and qRT-PCR. (.pdf, 69 KB)
