# Supplementary material for: Mutations in Dnaaf1 and Lrrc48 Cause Hydrocephalus, Laterality Defects, and Sinusitis in Mice
Source: G3 (Bethesda). 2016 Jun 3;6(8):2479–87. doi: 10.1534/g3.116.030791 (PMC4978901; doi:10.1534/g3.116.030791)
Supplement: Supplemental Material [file supp_g3.116.030791_FigureS2.pdf]

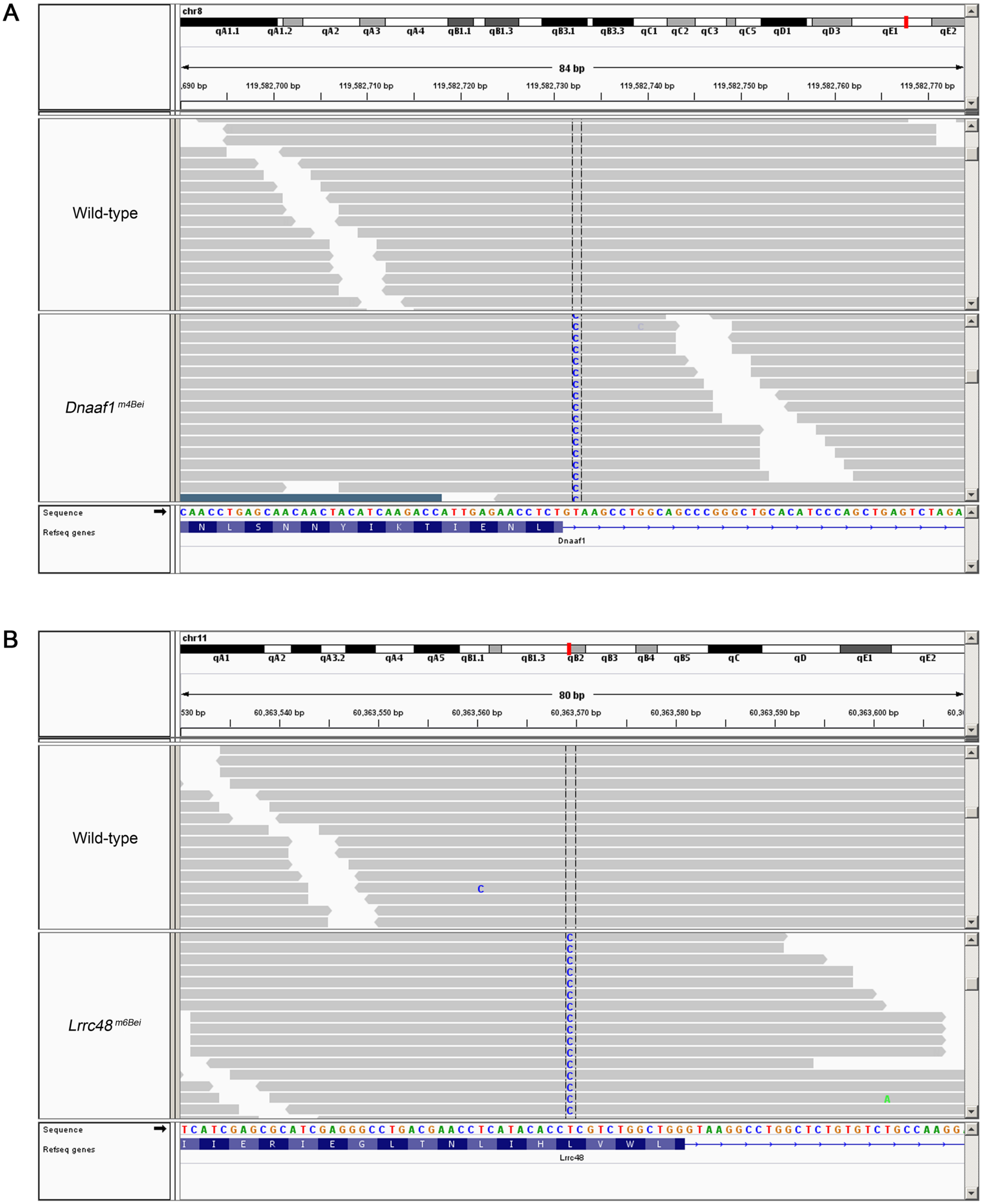


**Figure S2** Exome sequencing results. Snapshots of the integrated genome viewer (IGV) are showing mutations in (A) *Dnaaf1*$^{m4Bei}$ and (B) *Lrrc48*$^{m6Bei}$.
