# Supplementary material for: Mutations in Dnaaf1 and Lrrc48 Cause Hydrocephalus, Laterality Defects, and Sinusitis in Mice
Source: G3 (Bethesda). 2016 Jun 3;6(8):2479–87. doi: 10.1534/g3.116.030791 (PMC4978901; doi:10.1534/g3.116.030791)
Supplement: Supplemental Material [file supp_g3.116.030791_FigureS3.pdf]

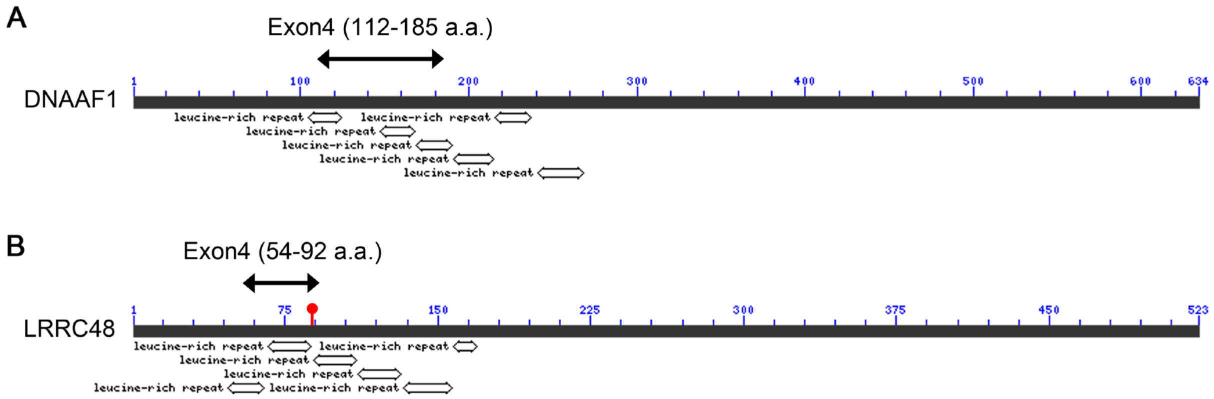

Figure S3 Protein domain structures from NCBI conserved domain search. (A) DNAAF1, 634 amino acids. Double-headed arrow (filled black) indicates a region corresponding to Exon 4. (B) LRRC48, 523 amino acids. Double-headed arrow (filled black) indicates a region corresponding to Exon 4, including the 89th amino acid leucine (red bulb). Locations of the leucine-rich repeat domains are indicated as Double-headed arrow. Both mutations are within the leucine-rich repeat domain.
