# Supplementary material for: Mutations in Dnaaf1 and Lrrc48 Cause Hydrocephalus, Laterality Defects, and Sinusitis in Mice
Source: G3 (Bethesda). 2016 Jun 3;6(8):2479–87. doi: 10.1534/g3.116.030791 (PMC4978901; doi:10.1534/g3.116.030791)
Supplement: Supplemental Material [file supp_g3.116.030791_FigureS4.pdf]

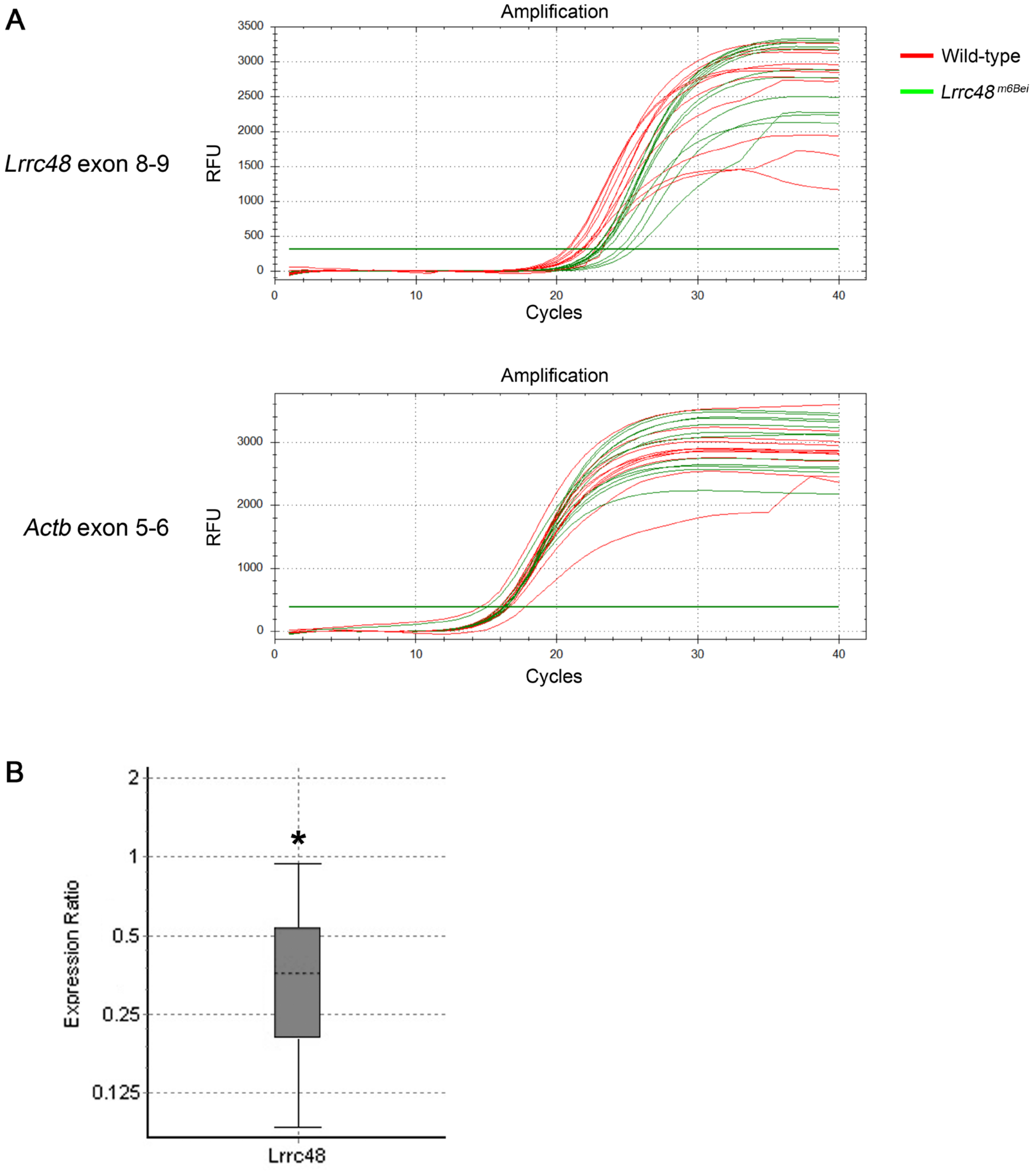

Figure S4 qRT-PCR analysis of *Lrrc48*. (A) Amplification curves of *Lrrc48* and *Actb* from qRT-PCR analysis. A shift of the green lines (mutants) in *Lrrc48* amplification curve to right compared to red lines (wild-type) is evident. (B) A graph showing REST analysis result of qRT-PCR data shown in A. *Lrrc48* ^{m6Bei} transcript expression ratio (0.335, P<0.001) of the mutant compared to the wild-type is plotted. The box represents the middle 50% of observations, the dotted line represents the median, and the whiskers represent the minimum and maximum observations.
