# Supplementary figures and images for: Mutations in Dnaaf1 and Lrrc48 Cause Hydrocephalus, Laterality Defects, and Sinusitis in Mice
Source: G3 (Bethesda). 2016 Jun 3;6(8):2479–87. doi: 10.1534/g3.116.030791 (PMC4978901; doi:10.1534/g3.116.030791)

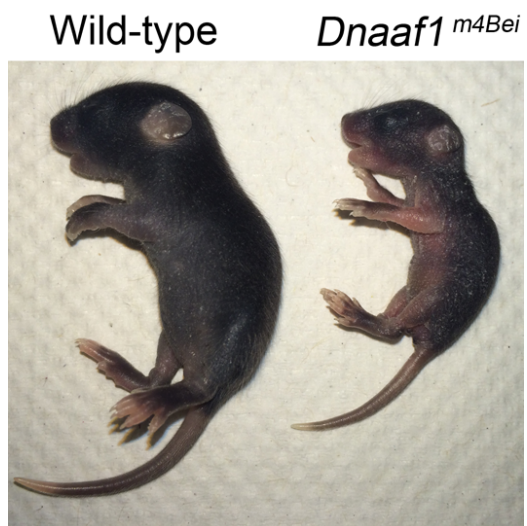

**Figure S5** *Dnaaf1*<sup>m4Bei</sup> homozygotes are runted. P7 pups are shown.

Supplement: Supplemental Material [file supp_g3.116.030791_FigureS5.pdf]
