# Supplementary material for: Mutations in Dnaaf1 and Lrrc48 Cause Hydrocephalus, Laterality Defects, and Sinusitis in Mice
Source: G3 (Bethesda). 2016 Jun 3;6(8):2479–87. doi: 10.1534/g3.116.030791 (PMC4978901; doi:10.1534/g3.116.030791)
Supplement: Supplemental Material [file supp_g3.116.030791_TableS1.pdf]

Table S1 Primers used for genotyping.

***Dnaaf1* ^{m4Bei} genotyping primers**			
Primer pair	Primer 1	Primer 2	Product size[a]
D8Mit132	ATTTGTTTTGCTTACTTCTCAGTGT	AGAATGAAGATATCCAGAGATGCC	84 bp/100 bp (WT[b]/MU[c])
D8Mit242	TGTGCAACCAATTTCTTCCA	CCCATGATTTATTCAGACTGAGG	166 bp/190 bp (WT/MU)
D8Mit280	CATGCAATTCCAATGTCAGTG	TAGCACTCAATCAAACCCCC	108 bp/160 bp (WT/MU)
Dnaaf1 e4-5	TGGCATCCAGAGGATCGAGA	CTCAATGTCAGCCACCGTCT	342 bp (160th T>C)
***Lrrc48* ^{m6Bei} genotyping primers**			
Primer pair	Primer 1	Primer 2	Product size
D11Mit20	CCTGTCCAGGTTTGAGAGGA	CTTGGGAGCCTCTTCGGT	116 bp/150 bp (WT/MU)
D11Mit310	GTGCACTTTCCATGCCTGTA	GAGTAGAAAGAGACAGAGAAAGACACA	126 bp/132 bp (WT/MU)
D11Mit298	AAACAAACAAAAATGCACCTCA	GTACCACCATGCCTAGCCTC	195 bp/221 bp (WT/MU)
Lrrc48 e4-i4	ACATCCTCCGCATCGATAAC [d]	CGTGAACCCAGAAGCCTCTT	494 bp (106th T>C)

a. Product sizes are found from MGI database.

b. WT (wild-type) indicates C57BL/6 allele.

c. MU (mutant) indicates A/J allele.

d. Used for sequencing.
