# Supplementary material for: Mutations in Dnaaf1 and Lrrc48 Cause Hydrocephalus, Laterality Defects, and Sinusitis in Mice
Source: G3 (Bethesda). 2016 Jun 3;6(8):2479–87. doi: 10.1534/g3.116.030791 (PMC4978901; doi:10.1534/g3.116.030791)
Supplement: Supplemental Material [file supp_g3.116.030791_TableS2.pdf]

Table S2 Primers used for RT-PCR and qRT-PCR.

Dnaaf1 ^{m4Bei} **RT-PCR primers (Figure 1B)**			
Primer pair	Forward primer	Reverse primer	Product size
Dnaaf1 e3-6	CCCTGAACGACACCCTGTAT	TGCTTCAGACGGACAGTGAC	501 bp
Lrrc48 ^{m6Bei} **RT-PCR primers (Figure 1E)**			
Primer pair	Forward primer	Reverse primer	Product size
Lrrc48 e2-3	AGCTTTCTGCTGCTTCTCCA	CTGCAGGGACAGGACATCTT	245 bp
Lrrc48 e4-7	ACATCCTCCGCATCGATAAC	ACTCCTCTGCCTCAGAAACG	360 bp
Lrrc48 e11-13	TCACTGACTTGGTGGGACTATTT	TGATTCCAGTCACCAGCTCA	270 bp
Actb e3-4	GGTGGGAATGGGTCAGAAGG	GCACGATTTCCCTCTCAGCT	497 bp
Lrrc48 ^{m6Bei} **qRT-PCR primers (Figure S4)**			
Primer pair	Forward primer	Reverse primer	Product size
Lrrc48 e8-9	GGGCAACAAGCTGTCCTACCTG	GGCCTCCTGTATGCACTCATTG	160 bp
Actb e5-6	AGATCAAGATCATTGCTCCTCCTG	AGCTCAGTAACAGTCCGCCT	170 bp
