# Supplementary material for: Mutations in Dnaaf1 and Lrrc48 Cause Hydrocephalus, Laterality Defects, and Sinusitis in Mice
Source: G3 (Bethesda). 2016 Jun 3;6(8):2479–87. doi: 10.1534/g3.116.030791 (PMC4978901; doi:10.1534/g3.116.030791)
Supplement: Supplemental Material [file supp_g3.116.030791_FigureS1.pdf]

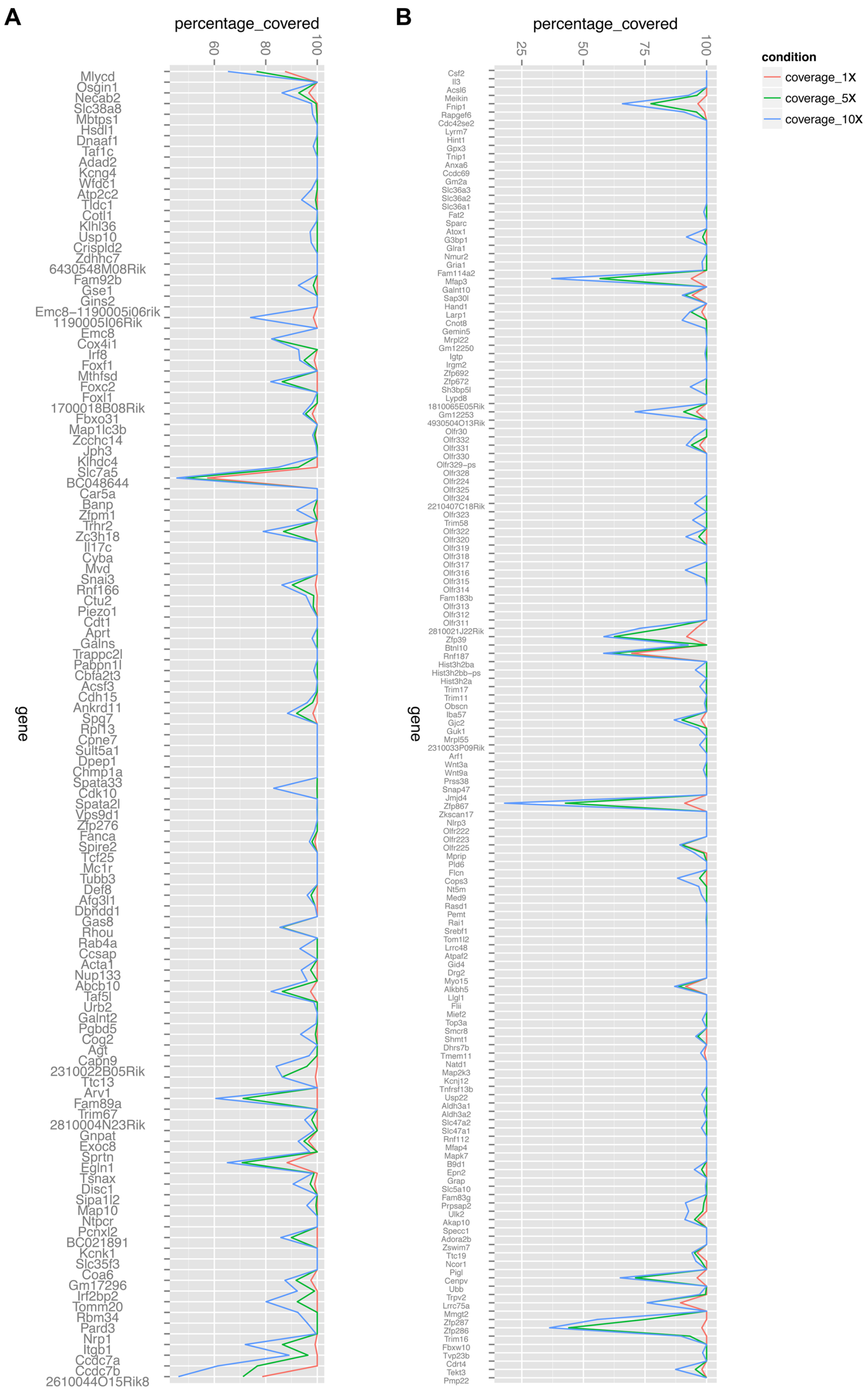

Figure S1 Lists of genes aligned against exome sequencing coverage. Percentage of bases with 1X, 5X, and 10X coverage are plotted. (A) *Dnaaf1* ^{m4Bei} and (B) *Lrrc48* ^{m6Bei}.
